# The Value of Local Treatment in Syphilitic Ulceration of the Larynx and Pharynx

**Published:** 1881-06

**Authors:** J. D. Arnold

**Affiliations:** Baltimore


### THE VALUE OF LOCAL TREATMENT IN SYPHILITIC ULCERATION OF THE LARYNX AND PHARYNX.

By J. D. ARNOLD, M.D., BALTIMORE.

Although fully aware of the almost universal scepticism as regards the efficacy of topical treatment in any of the varied manifestations of syphilis upon the mucous surfaces, I shall venture to submit a few considerations in this connection, and my excuse for asking attention to a subject upon which so much has already been said, is that during a six months' attendance at the Vienna Clinic, I have taken special care to observe and note the results of the several methods of treatment there in use.

The importance of bringing about a quick process of healing in syphilitic ulcerations of the pharynx and larynx is evident not only from the readiness with which their delicate structures yield to the inroads of this disease, but especially because of the considerable impairment of voice, which comparatively slight loss of substance in these regions so often occasions. In the pharynx, for instance, just that structure—the soft palate, which is most intimately concerned in giving timbre and resonance to the voice—is the favorite seat of specific ulceration, and being of a loose, spongy nature, offers little resistance to its destructive changes. It is certainly true that this disease in the pharynx and larynx is only a local manifestation of a systemic poison for which we possess specifics in iodine and mercury; still it very often happens that before the exhibition of these drugs produces an impression upon the system at large, such changes have taken place in the organ of voice as to impair it irreparably.

Although the views of Sigmond, who insisted that constitutional remedies are neither indicated nor valuable in syphilitic affections, unless some broad constitutional symptoms be present, have been entirely abandoned by the majority of later syphilographers, distinguished names are not wanting amongst the exponents of his teachings, even at the present day. Morrell Mackenzie, in the introduction to his late book on "Diseases of the Larynx," asserts himself to be in a great measure a follower of Sigmond, as regards the relative merits of general and local medication in a large class of venereal troubles, especially those of the respiratory tract. At all events, any means by which the breaking down of tissue may be prevented or held in check until constitutional remedies shall have become operative, must certainly be valuable and desirable aids to treatment. That such means are ready at hand I am convinced from the close observation of their use in a large number of cases of specific ulceration in the throat.

The syphilitic ulcer in the pharynx and larynx is nearly always due to the breaking down of the papule which runs exactly the same course upon the mucous membrane as upon the skin. It first distinguishes itself as a small nodule raised upon the surrounding surface, has a dry, glistening appearance due to the stretching of its epithelial covering by the cell infiltration beneath, and presents a dark brownish-red color. From this nucleus the infiltration extends circularily; the oldest portion of the papule (the centre) breaking down first, loses its epthelia and exposes a raw ulcerating surface. When the ulcer has once formed, it rapidly spreads, and its secretions, which are chiefly composed of epthelial detritus and pus cells, contains highly infectious properties. The edges of the ulcer, whether they be round or irregular, are always bounded by a deep red halo, probably caused by the escape of the coloring matter of the blood from compression of the vessels by cell infiltration. In those cases where doubt exists between syphilis and tuberculosis, this red margin of infiltration is a valuable aid to diagnosis, the tuberculous ulcer being nearly always surrounded by pale anæmic mucous membrane.

The vocal chords are not infrequently the seat of ulceration, which usually spreads longitudinally upon their surfaces, and makes their vibrating edges ragged and uneven. In this region the loss of substance is often so great that the vocal processes of the arytenoids are laid bare; in such cases phonation becomes imperfect and the voice remains forever hoarse. When the ventricular bands or arytenoids are attacked by the syphilitic process an œdema is occasionally developed, which does not readily yield to the ordinary measures for the relief of this complication. It goes without saying that the greater the destruction of tissue before the ulceration is checked, the more considerable will be the distortion of parts from subsequent cicatrization.

In the pharynx, as before stated, the parts most frequently attacked by specific ulcer are the soft palate and uvula, and since, for the articulation of vowels and vocals it is necessary that the naso-pharynx shall be cut off from the mouth cavity, it will readily be seen that any perforation or loss of contiguity in the velum must greatly interfere with distinct speech. The most notable examples of defective speech from an analogous cause, are cases of paralysis of the soft palate, after diphtheria. It is quite common that the posterior portion of the velum palate is the seat of an ulcer, and during the process of healing becomes adherent to the wall of the pharynx; in this manner the mouth is permanently cut off from the superior pharyngeal cavities, and in consequence the voice remains nasal and muffled.

Without regarding, therefore, the more serious complications which may arise from syphilis in the larynx and pharynx, it is plain that the integrity of speech and voice is much endangered by this destructive process whose speedy abortion is only possible by a prompt and active treatment.

As far as the general anti-syphilitic treatment is concerned, the choice between iodine and mercury must in some measure be influenced by each individual case; but experience seems to show that when dealing with ulceration upon mucous surfaces mercury by inunction makes a quicker and more decided impression than any other method of medication. Although, as I mention above, the papule is the precursor of throat ulceration in the great majority of instances, gummata do sometimes occur as manifestations of the later or tertiary forms. In these cases the iodide of potassium or sodium is to be preferred to mercury. In this form of the disease every effort must be made to produce a rapid impression with the drug, and to this end large doses must be given, 6-12 grammes in twenty-four hours. To this small quantities of mercury bichloride may be added with advantage. Of course as soon as the iodine gives positive evidence of its presence in the system, the dose must be reduced to the medium. The importance of a good nourishing diet cannot be too much insisted upon during the course of any specific treatment.

Nearly all writers upon the subject devote very little space to the local treatment of syphilitic sore throat, and after giving a meagre list of astringent washes and sprays wherewith the ulcerating surfaces may be properly cleansed, they almost invariably end their short chapter of thereapeutics with the naive remark that after all very little must be expected from topical medication. The use of iodiform, which has been lately much extolled, is not well tolerated in the larynx or pharynx, because of its highly disagreeable odor, and I have seen no decided benefit from its exhibition. The method of local treatment which is employed at the Vienna Clinic, and from which I have seen the most satisfactory results, consists in pencilling the face of ulceration with the following solution:

| | | |
|---|---|---|
| R. | Iodini Potassæ, - - - - - | 0.5 |
| | Iodini Purac - - - - - | 0.3 |
| | Glycerini, - - - - - - | 40.00 |

The diseased surface having been thoroughly cleansed of all secretion by careful washing with a weak solution of copper sulphate, the iodo-glycerine is applied with the brush *two* or *three* times *daily*, whilst the infiltrated edge of the ulcer is occasionally touched with silver nitrate upon a metal sound.

I transcribe from my notes of a large number treated in this manner, a synopsis of four cases, which I have chosen because I think they fairly illustrate the point that a properly directed local treatment will effect, or very materially aid, the healing of syphilitic ulcers in the throat and mouth.

*Case I.*—A girl, aged 17, came to clinic November 4th. Complained of hoarseness, cough and pain in swallowing. Laryngoscopic examination discovered a small irregular ulcer upon the posterior portion of right vocal cord, slightly encroaching upon the arytenoid of same side. Surface of ulcer covered with grayish-white exudation. Mucous covering of pharynx reddened and engorged, a few isolated follicles enlarged. The patient had, in addition to the throat trouble, a papular syphilide upon face, neck, arms and breast. The ulcer in larynx was cleansed and brushed with iodo-glycerine, and the enlarged follicles were touched with solid silver nitrate. Patient was sent to syphilis ward with instructions to use three grammes ung. cin. by inunction daily, and to present herself for local treatment twice every day.

Nov. 6th. Ulcer on cord has not spread since the beginning of treatment, and has a cleaner smoother appearance. Edges of infiltration touched with fused silver nitrate.

Nov. 9th. The patient declares that her throat is much better, as she experiences no difficulty in swallowing. Examination with mirror shows that repair has already commenced. There is no longer a well-marked border of infiltration; the surrounding healthy tissues seem to send their projections into the diseased portion of the cord. (These thin white glistening bands are the first cicatricial formations, and evidence the process of healing).

Patient is still somewhat hoarse. The eruption upon the skin shows as yet no signs of subsiding.

Nov. 14th. The ulcer has entirely disappeared, leaving no traces except a thin cicatricial membrane, which slightly impedes the finer movements of the cord. Eruption on skin as before.

Nov. 22d. The former seat of ulceration in larynx is scarcely distinguishable; voice normal; papular syphilide on skin somewhat paler.

*Case II.*—A hostler, aged thirty-one, came to clinic November 13th, complaining of pain in swallowing and great soreness in mouth Inspection of pharynx showed a broad shallow ulceration upon the right pharyngo-palatine arch. The mirror discovered a small round ulcer upon the superior edge of the epiglottis. Patient admitted having had a chancre two years ago. He was stripped, and a grouped small-papular eruption found upon his breast and abdomen. He could not enter the hospital, and was treated as an ambulant. Ulcerations upon soft palate brushed with iodo-glycerine, ungt. cin. ad. gammes 3, inunction daily.

Nov. 17th. The ulcer on epiglottis has entirely disappeared; that upon velum somewhat contracted and free from detritus. Eruption on skin spreading; a few spots on arms and thighs.

Nov. 23d. Ulcer on soft palate completely healed; a thin cicatrix draws the uvula somewhat to the right side. Eruption on skin unchanged. (This patient continued inunction until December 19th, at which time the syphilide had completely faded )

*Case III.*—A strong, healthy-looking woman, 38 years old, applied to clinic October 6th, with a deep ulcer on base of tongue extending on to left gloso-pharyngeal fold. All other districts of the mouth, pharynx and larynx normal in appearance. Denies ever having had syphilis. Put upon 5 grammes potassæ iodide daily; no local medication.

Oct. 9th. Ulcer on tongue has spread to left pharynx wall, and two small erosions have made their appearance on the internal face of the epiglotis Iodide increased to ten grammes daily.

Oct. 14th. No perceptible change in ulcer on tongue and pharynx; erosions on epiglottis have developed into an irregul r ulcerating surface covered with thick white secretion. Iodide stopped. Ung. cin. 3 grammes.

On Oct. 19th, although no considerable alteration had taken place in the ulcerations, the epiglottis had grown so thick and œdematous as to give rise to an alarming dyspnœa. Ice poultice was applied to the throat, and the ulcers for the first time pencilled with iod-glycerine.

On Oct. 20th the œdema had entirely subsided, and the breathing was quite easy. The ulcers on tongue and pharynx have a clean, red look, and there is hardly a trace of pus in their secretions.

Oct. 25th. Ulcer on epiglottis completely cicatrized.

Nov. 1st. Ulceration on tongue and pharynx entirely healed, with the exception that in the furrow in front of the glosso-pharyngeal fold (which has lost considerable of its substance) there is a small sluggish sore. Touched with galvano-cautery.

Nov. 6th. Patient was dismissed quite cured.

*Case IV.*—A boy, 16 years old, came to clinic February 10th, with a large ulcer covering nearly the whole of the soft palate. He states that he has been treated for three weeks, taking four pills a day (probably iodide ) Admitted to the ward and treatment immediately commenced with pencillings of iod-glycerine.

On Feb. 23d he was dismissed cured, with no other evidence of the former enormous ulcer than that the velum was covered with thin, scarcely perceptible stellate cicatrices and presented immediately over the centre of its left arch a small perforation that would, with difficulty, admit the point of a lead pencil.

I think that in the first two cases the healing of the ulceration may in great part be fairly ascribed to the use of the iod-glycerine, for in both cases the throat trouble subsided long before the skin eruption showed any signs of disappearing, whereas in the absence of local treatment this order is usually reversed. In case number three the recourse to the brush was followed by such immediate improvement that there is no room for doubt as to the part it played. Case four was a trial of the iod glycerine without any constitutional medication, and although the result was a happy one, it does not justify the conclusion that in such cases general treatment is unnecessary.—*Maryland Medical Journal.*

